# Simplified Centralization Technique With Dual-Tunnel Meniscal Root Repair

**DOI:** 10.1016/j.eats.2025.103709

**Published:** 2025-07-05

**Authors:** Justin Jabara, Marcus Trotter, Jamie E. Confino, Elly LaRoque, Nicholas Colyvas

**Affiliations:** Department of Orthopaedic Surgery, University of California, San Francisco, San Francisco, California, U.S.A.

## Abstract

Meniscal extrusion has been identified as a major risk factor for altered knee kinematics, aberrant forces, and potential osteoarthritic progression. Failure of the meniscotibial ligament complex appears to be the primary cause. Centralization techniques have been developed to help properly reduce the extruded meniscus to its footprint, which can potentially restore its function in dissipating hoop stresses. Many described centralization procedures use suture anchors with an accessory portal, which can be somewhat technically challenging. We present our simplified meniscal centralization technique using all-inside meniscal repair devices to repair the meniscotibial ligament, reducing it to its footprint using a transtibial tunnel.

The menisci have been recognized as vital intra-articular structures that are critical for knee function and articular preservation. They act as shock absorbers and force transmitters, provide lubrication, and serve as secondary stabilizers to anterior tibial translation.^1^, ^2^, ^3^, ^4^ There has been a trend to expand repair indications in an attempt to preserve the meniscus, which could limit the sequelae of pain, instability, and arthritis.^5^

Recent clinical investigation has focused on meniscal extrusion and its role in inciting articular damage and subsequent degenerative joint disease.^6^^,^^7^ Meniscal extrusion is defined as pathologic displacement of the meniscus outside of the tibial plateau margins.^8^ Historically, extrusion greater than 3 to 4 mm, or greater than 30% of the meniscal width, was defined as pathologic and considered to warrant further attention.^8^, ^9^, ^10^ Although some evidence suggests that extrusion is partly a physiological response, there is even more evidence that it represents a pathologic consequence seen with radial and root tears, among others.^11^^,^^12^

Disruption of the meniscotibial ligament has been identified as a primary etiology for extrusion among other factors such as body mass index, knee alignment, and age.^2^^,^^13^, ^14^, ^15^ Whether extrusion occurs first, predisposing the meniscus to increased forces and failure, or occurs after a meniscal tear is being debated.^16^ Indications for centralization are also being debated. Nevertheless, it is important to pay intraoperative attention to extrusion. Normalizing the anatomy of the meniscus aids its ability to withstand hoop stresses and protect articular cartilage from excessive forces.^2^^,^^15^^,^^16^

Studies have shown that residual extrusion can present after meniscal repair.^17^^,^^18^ A number of centralization techniques have been developed to better reduce the extruded meniscus to its anatomic footprint. Options such as anchoring the meniscus to the adjacent tibial plateau surface using all-suture or knotless anchors, deep medial collateral ligament repair, meniscotibial repair, and suture augmentation of the meniscus have been described for meniscal centralization.^19^, ^20^, ^21^, ^22^ The current technique uses all-inside meniscal repair devices to repair the meniscus at the location of the meniscotibial ligament, which is then reduced to its footprint using a distinct transtibial tunnel.

## Surgical Technique

**Fig 1:**
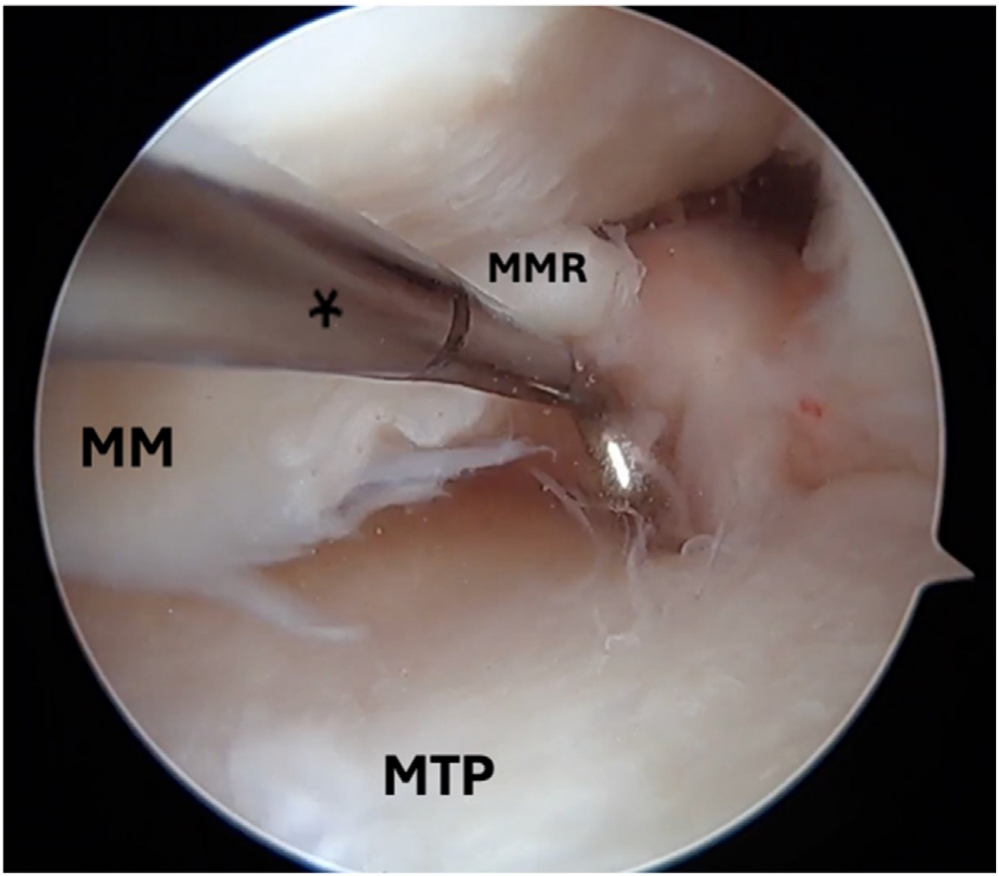
An intraoperative arthroscopic photograph, viewing from a standard anterolateral portal with a 30° arthroscope, shows a left medial meniscal root tear during diagnostic arthroscopy. An arthroscopic probe (asterisk) is introduced from a standard anteromedial portal to help assess the meniscal root. (MM, medial meniscus; MMR, medial meniscal root; MTP, medial tibial plateau.)

**Fig 2:**
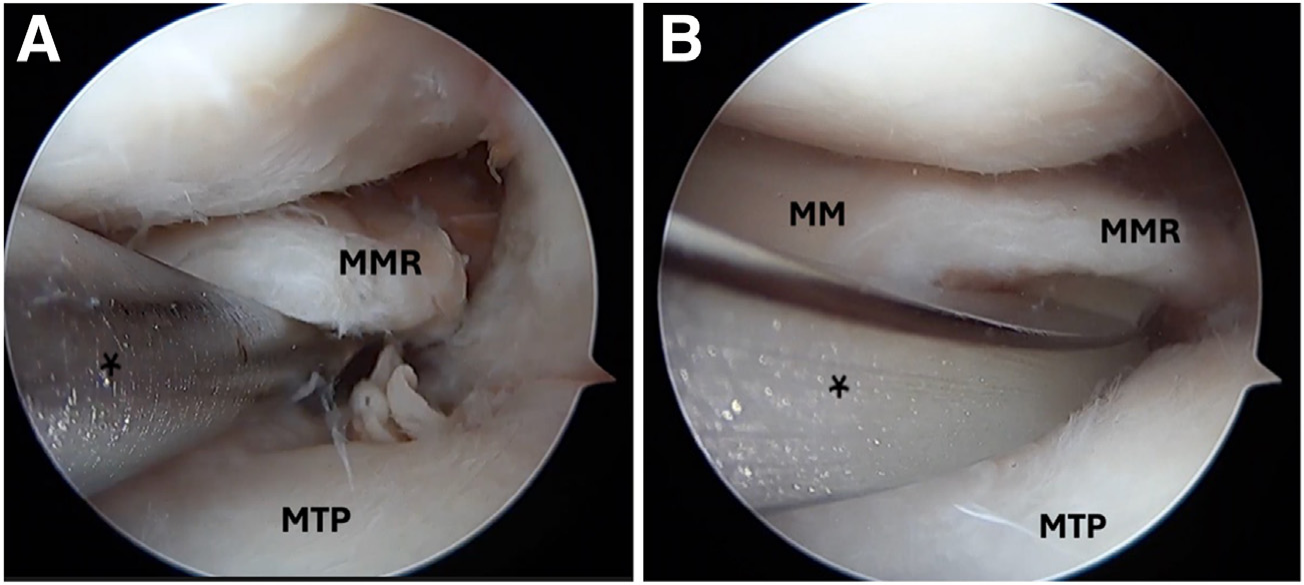
Intraoperative arthroscopic photographs left knee viewing from a standard anterolateral portal with a 30° arthroscope. (A) A curette (asterisk) is used to create a socket at the proposed root tunnel aperture. (B) A meniscal root guide (asterisk) (Arthrex) is used to create a tibial tunnel to the proposed root socket through the anteromedial portal. (MM, medial meniscus; MMR, medial meniscal root; MTP, medial tibial plateau.)

**Fig 3:**
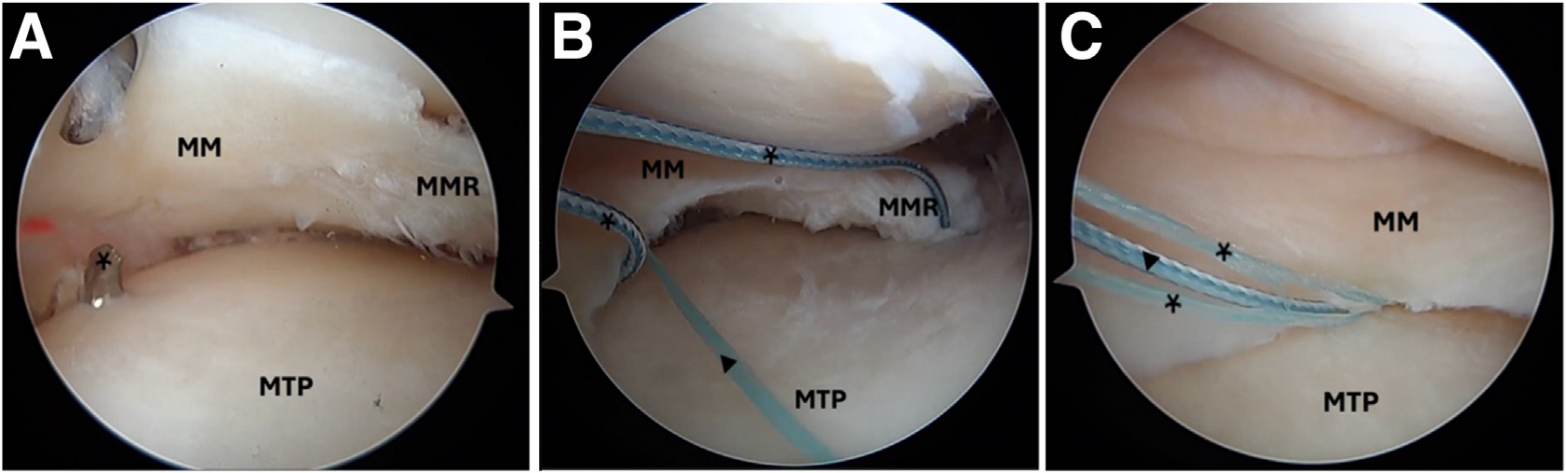
Intraoperative arthroscopic photographs left knee viewing from a standard anterolateral portal with a 30° arthroscope. (A) The second centralization tunnel (asterisk) is created at the medial aspect of the tibial plateau using a similar technique to that used for the first tunnel. The outside aperture of the centralization tunnel is approximately 2 to 3 cm medial to the root tunnel and in line with the posterior border of the medial collateral ligament, where maximum extrusion typically occurs. (B) Shuttling sutures (asterisks) are passed through both tunnels. Two all-inside meniscal repair implants (triangle) (AIR) are then placed at the meniscotibial ligament, inferior to the meniscus, from the anterolateral portal. (C) The tightening suture tails (asterisks) are not cut and are shuttled through the centralization tunnel using the shuttling suture (triangle). (MM, medial meniscus; MMR, medial meniscal root; MTP, medial tibial plateau.)

**Fig 4:**
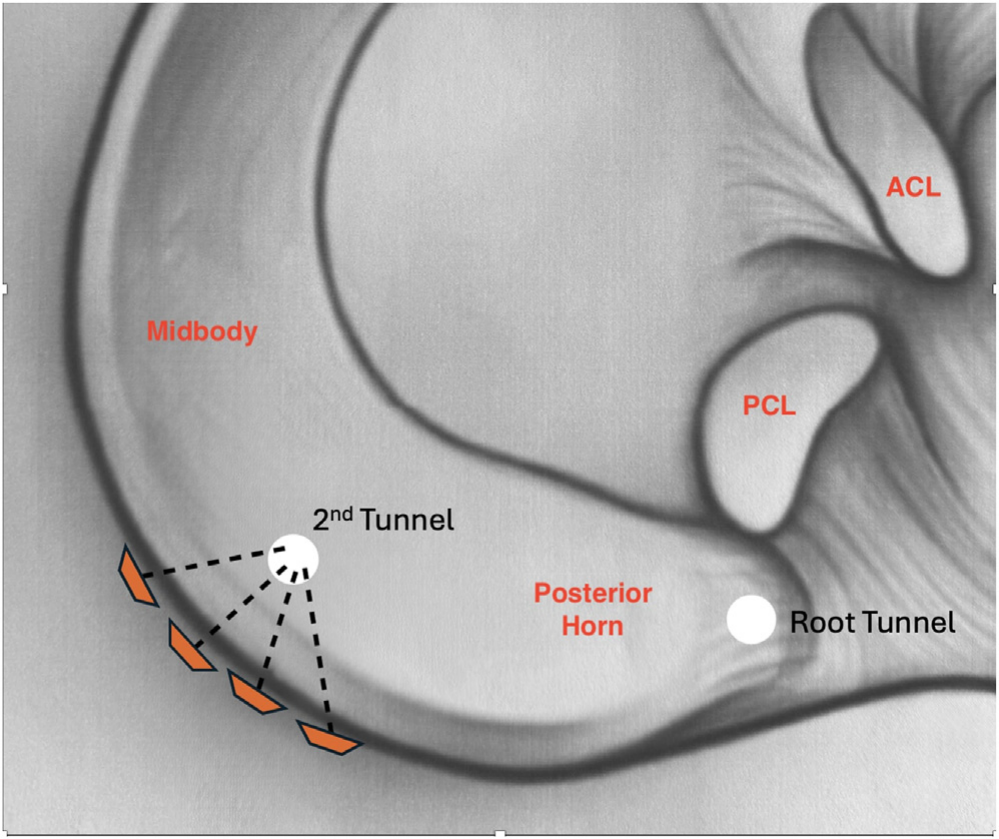
Arrangement of tunnels and meniscal repair devices in relation to posterior horn of medial meniscus. (ACL, anterior cruciate ligament; PCL, posterior cruciate ligament.)

Standard anterolateral and anteromedial arthroscopic knee portals are created. The procedure begins with diagnostic arthroscopy using a 30° arthroscope from the anterolateral portal. A radial medial meniscal root tear with extrusion is identified (Fig 1), and the frayed edges are debrided with a shaver. The decision is made to perform a dual-tunnel repair (Video 1). A curette is used to create a socket at the proposed root tunnel aperture (Fig 2A). A 3-cm incision is made at the anteromedial tibia, and a meniscal root guide (Arthrex, Naples, FL) is used to create a tibial tunnel to the proposed root socket (Fig 2B). A 2.4-mm drill tip guide pin is used, followed by a 4.5-mm cannulated reamer. The reamer head is retained in the joint with the tip visible, the guide pin is removed, and a shuttle suture (FiberStick; Arthrex) is passed through the cannulated reamer and retrieved with a grasper through the anteromedial portal. The second tunnel is created using a similar technique at the medial aspect of the tibial plateau (Fig 3A). The aperture of the centralization tunnel is approximately 2 to 3 cm medial to the root tunnel and in line with the posterior border of the medial collateral ligament, where extrusion is typically maximal (Fig. 4). A second shuttling suture is passed in the same fashion (Fig 3B). A curved curette is then used to prepare the footprint for the meniscotibial ligament repair at the far periphery of the plateau over the tunnel aperture. Two all-inside meniscal repair implants (AIR; Stryker, Kalamazoo, MI) are placed at the meniscotibial ligament, inferior to the meniscus, from the anterolateral portal. These are placed broadly to capture a significant portion of the meniscotibial ligament to tension it against the prepared tibial surface. The tightening suture tails are not cut and are shuttled through the centralization tunnel (Fig 3C).

**Fig 7:**
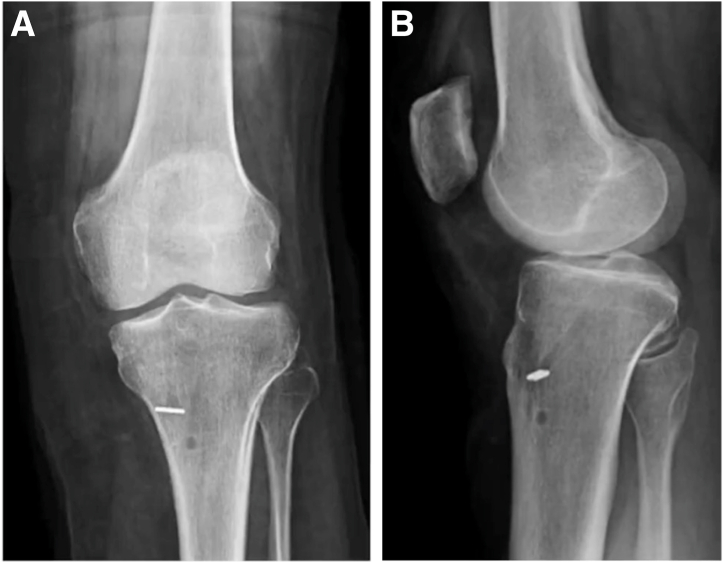
(A, B) Postoperative radiographs left knee after medial meniscal root repair with centralization.

**Fig 5:**
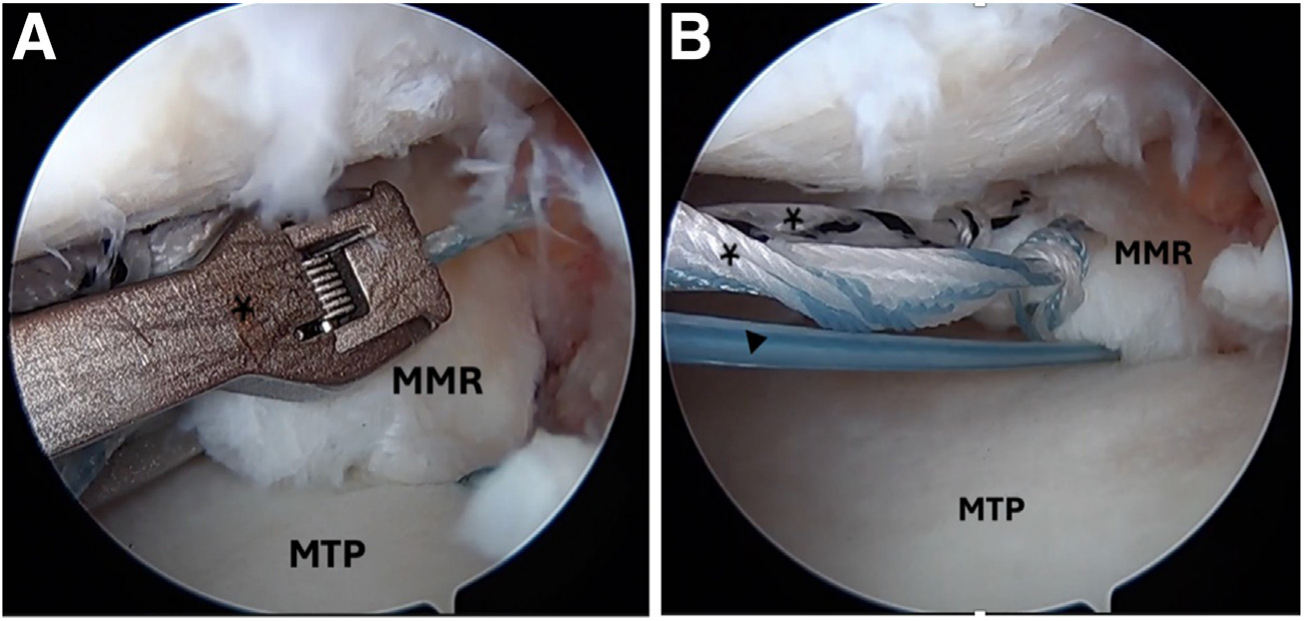
Intraoperative arthroscopic photographs left knee viewing from a standard anterolateral portal with a 30° arthroscope. (A) A self-capturing suture passer (asterisk) (Knee Scorpion) is used to pass two 1.3-mm suture tapes in a luggage-tag configuration at the meniscal root. (B) The 2 sutures (asterisks) are shuttled through the root tunnel using the shuttling suture (triangle). (MM, medial meniscus; MMR, medial meniscal root; MTP, medial tibial plateau.)

**Fig 6:**
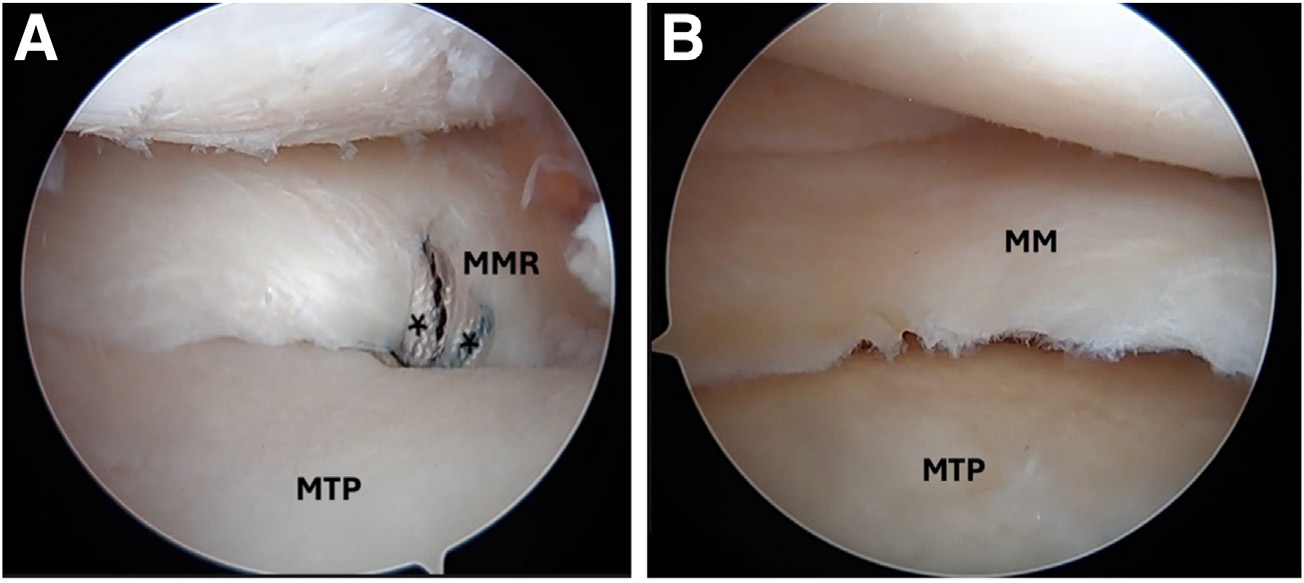
Intraoperative arthroscopic photographs left knee viewing from a standard anterolateral portal with a 30° arthroscope. Both the medial meniscal root sutures (asterisks) (A) and centralization sutures (B) are tensioned appropriately and then tied over a 15-mm titanium cortical button (Rigidloop). Backup fixation is performed distal to the cortical button with a 4.75-mm knotless suture anchor (Argo). (MM, medial meniscus; MMR, medial meniscal root; MTP, medial tibial plateau.)

A standard root repair is then performed. A self-capturing suture passer (Knee Scorpion; Arthrex) is used to pass two 1.3-mm suture tapes in a luggage-tag configuration at the meniscal root (Fig. 5A). These are subsequently shuttled through the root tunnel (Fig. 5B). Both sets of sutures are tensioned appropriately (Fig. 6) and then tied over a 15-mm titanium cortical button (Rigidloop; DePuy Mitek, Raynham, MA). Backup fixation is performed distal to the cortical button (Fig. 7) with a 4.75-mm knotless suture anchor (Argo; ConMed, Largo, FL). Microfracture of the notch is performed to further encourage meniscal healing.

### Postoperative Rehabilitation

The patient is instructed to remain non–weight bearing on the operative extremity for 6 weeks. A hinged knee brace is worn at all times and is locked in full extension except during range-of-motion exercises. For the first 6 weeks active non–weight-bearing extension exercises to maintain knee strength and reduce muscle atrophy are instituted. At rest, range of motion from 0° to 90° is encouraged. After 6 weeks, the patient is advanced to full weight bearing and range of motion.

## Discussion

There has been an increasing emphasis on meniscal root preservation, given its unique role in shock absorption and force dispersion, which is chondroprotective.^1^, ^2^, ^3^^,^^5^ As shown in the literature, biomechanically, a meniscal root tear behaves similarly to a total meniscectomy.^23^ Recently, meniscal extrusion has been the focus of significant investigation because it leads to increased articular forces, diminished patient outcomes, and progression to arthritis.^2^^,^^6^^,^^7^^,^^15^^,^^16^ Considering these unfortunate consequences, centralization techniques have evolved to address meniscal extrusion surgically while creating a stable environment for the meniscal repair to heal.

The described technique is unique for several reasons. Using an independent transtibial tunnel to address meniscal extrusion eliminates the need for an additional suture anchor and accessory portal. An all-inside meniscal repair device is used in this technique to capture the fibers of the meniscotibial ligament for reduction. These devices are easy to place and deploy. They also provide a broader capture of the meniscus than a simple meniscal suture would in this role. Because sutures are not placed around the meniscus and are only placed into the meniscotibial ligament complex, the risk of suture cut-through and a subsequent iatrogenic radial tear of the meniscus is eliminated. Given that the meniscotibial ligament plays a significant role in meniscal stability, we believe that incorporating this tissue into the repair is essential to address extrusion.^2^^,^^15^^,^^24^

The described technique is simple and reproducible, which offers multiple advantages. Only 1 tibial incision is needed to create both tunnels. Thus, an additional accessory portal, as described in other techniques, is not needed. Inaccurate placement of an accessory portal can theoretically damage the collateral ligaments, and this risk diminishes with the presented technique. Using 2 all-inside devices promotes sufficient fixation to the meniscotibial ligament by providing substantial strength from the No. 2-0 ultrahigh-molecular-weight polyethylene suture and adequate reduction to correct extrusion. Once the repair sutures are shuttled through their respective tunnels, intraoperative inspection of the repair can be performed to confirm adequate reduction with the sutures under tension. If suture anchors are used, the meniscal body reduction relies on adequate knot tying or activation of a knotless suture mechanism. There is potential for creep and loss of fixation with improper suture anchor placement, with an inadequate knot-tying technique, or if the knotless suture mechanism cannot fully activate owing to soft-tissue interposition. Adding an additional tunnel promotes direct blood and bone marrow application to the centralization site, potentially increasing mesenchymal stem cell recruitment to the area of repair. Microfracture of the intercondylar notch can also be performed to enhance biologic meniscal healing response.^25^ Finally, the root and centralization sutures are fixated on the tibia using only 1 cortical button and backup suture anchor, which limits costs (Table 1).

**Table 1:** Advantages and Disadvantages of Dual-Tunnel Meniscal Root Repair With Centralization

Advantages
One incision is used for both meniscal root repair and centralization.
Secure fixation of the meniscotibial ligaments is achieved using an all-inside device.
The transtibial technique allows direct compression and reduction of the midbody and meniscotibial ligaments to the anatomic footprint.
No additional fixation is needed because both the posterior root and midbody are fixated using the same cortical button and backup anchor.
A pericapsular incision is not warranted, which obviates the risk of inadvertent collateral ligament damage.
The additional tunnel further influences blood and mesenchymal stem cell migration into the joint and repair.
Sutures are not placed in or around the meniscus, reducing the risk of these cutting through the meniscus.
Disadvantages
Additional cost is incurred when using all-inside devices.
An additional tunnel is needed, leading to bone loss and a chance of tunnel convergence.
If ideal tunnel placement is not achieved, inaccurate reduction and residual extrusion can occur.
Peripheral tibial cortical blowout could occur if the aiming arm is placed too posterior.

There are disadvantages that must be considered with this technique. Using all-inside devices increases the cost of the procedure. There is a risk of the all-inside anchor protruding through the skin at the location of the meniscal midbody if placed without attention. An additional tunnel is needed, which increases the probability of tunnel convergence and bone loss while adding morbidity. Given that the tunnels are each 4.5 mm, we do not believe that bone loss or additive morbidity presents a major concern. Similarly to anchor placement described in other techniques, the tibial tunnel must be adequately placed. Improper placement can risk inappropriate reduction and the possibility of peripheral tibial cortical blowout (Table 1). Given that our technique is a 2-tunnel technique, it may present a learning curve to some surgeons. Multiple intra-articular sutures can increase the risk of soft-tissue bridging or entanglement and can increase operating room time to correct this issue. Further pearls and pitfalls can be reviewed in Table 2. This technique is reliable, is efficient, and provides consistency in reducing the meniscal root while correcting extrusion.

**Table 2:** Pearls and Pitfalls of Dual-Tunnel Meniscal Root Repair With Centralization

Pearls
Adequate fat pad debridement should be ensured for easy suture passage.
The surgeon should create adequate portals or consider cannula use to avoid tissue bridges and improve suture management; a suture retriever can be used to “run” sutures i.e. untangle them before shuttling.
An MCL pie-crusting technique allows improved visualization and easier passage of instruments, reducing the risk of iatrogenic articular damage and improving repair constructs.
Underlying cartilage and bone at the footprint should be removed with a curette to provide a “landing zone” for the meniscus while encouraging mesenchymal stem cell migration.
Before shuttling sutures through the tunnel, the surgeon should ensure there is no soft-tissue bridge.
The use of a guide pin prior to a reamer ensures confirmation of adequate tunnel placement.
The 4.5-mm cannulated reamer creates a low-profile tunnel, which minimizes bone loss and morbidity. Being cannulated, it also allows easy passage of a shuttle suture.
If it is difficult to obtain the correct trajectory for the centralization tunnel, the surgeon should consider an accessory AM portal or the AL portal as the working portal.
The placement of the centralization tunnel should be kept in mind; the surgeon should ensure it is proximal relative to the root tunnel to minimize convergence.
It is critical to ensure adequate reduction arthroscopically when sutures are tied over the button to confirm proper tension.
The backup anchor reliably stabilizes the construct while holding tension.
The all-inside devices should be deployed at a shorter length than typical, and it is vital to confirm that the device has not protruded out of the skin at the location of the meniscal midbody.
A lateral incision can be used for tunnel placement. If used, the surgeon should consider independent fixation for each tunnel.
Pitfalls
The technique may present a learning curve to surgeons given the use of 2 distinct transtibial tunnels.
Multiple intra-articular sutures can increase the risk of a soft-tissue bridge or suture entanglement.
There is a risk of tunnel convergence if the tunnels are placed too closely.
There can be difficulty in obtaining the correct trajectory of the centralization tunnel using the standard AM portal.
If the AL portal is used to obtain the proper trajectory for the centralization tunnel, a separate lateral tibial incision may be needed.
Failure to inspect the skin after deploying the all-inside device could theoretically result in the device anchor deploying outside of the capsule, residing on the skin.

AL, anterolateral; AM, anteromedial; MCL, medial collateral ligament.

## Disclosures

The authors declare the following financial interests/personal relationships which may be considered as potential competing interests: E.L. reports a consulting or advisory relationship with Moximed. N.C. reports a consulting or advisory relationship with Convergence Surgical Robotics. All other authors (J.J., M.T., J.E.C.) declare that they have no known competing financial interests or personal relationships that could have appeared to influence the work reported in this paper.

## Supplementary Data

Video 1 Medial meniscal root repair using a dual-tunnel technique for meniscal centralization. The procedure begins with diagnostic arthroscopy of the left knee, confirming a meniscal root tear with extrusion. The frayed edges of the torn meniscal root are debrided with a shaver. A curette is then used to create a socket at the aperture of the proposed root tunnel. An incision is made at the anteromedial tibia, and a meniscal root guide (Arthrex) is used to create a tibial tunnel. A 2.4-mm drill tip guide pin is used through the guide, followed by a 4.5-mm cannulated reamer. The reamer is retained within the joint, with the tip visible, and the guide pin is removed. A shuttling suture (FiberStick) is passed through the cannulated portion of the reamer. A second tunnel is created using the same guide, at the medial aspect of the tibial plateau, in line with the posterior border of the medial collateral ligament. A second shuttling suture is passed. A curved curette is used to prepare the footprint for the meniscotibial ligament repair at the far periphery of the tibial plateau. Two all-inside meniscal repair implants (AIR) are then placed at the meniscotibial ligament, inferior to the meniscus. These are placed broadly to capture a significant portion of the meniscotibial ligament, tensioning it against the prepared tibial surface. The tightening suture tails are not cut; rather, they are shuttled through the centralization tunnel. Root repair is then performed. A self-capturing suture passer (Knee Scorpion) is used to pass two 1.3-mm suture tapes in a luggage-tag configuration, which are shuttled through the root tunnel. After tensioning, they are tied over a 15-mm titanium cortical button (Rigidloop). Backup fixation is performed distal to the cortical button with a 4.75-mm knotless suture anchor (Argo). Microfracture of the notch is then performed.
